# Repetitive Intermittent Hyperglycemia Drives the M1 Polarization and Inflammatory Responses in THP-1 Macrophages Through the Mechanism Involving the TLR4-IRF5 Pathway

**DOI:** 10.3390/cells9081892

**Published:** 2020-08-12

**Authors:** Fatema Al-Rashed, Sardar Sindhu, Hossein Arefanian, Ashraf Al Madhoun, Shihab Kochumon, Reeby Thomas, Sarah Al-Kandari, Abdulwahab Alghaith, Texy Jacob, Fahd Al-Mulla, Rasheed Ahmad

**Affiliations:** 1 Immunology & Microbiology Department, Dasman Diabetes Institute, Kuwait City 15462, Kuwait; hossein.arefanian@dasmaninstitute.org (H.A.); shihab.kochumon@dasmaninstitute.org (S.K.); Reeby.Thomas@dasmaninstitute.org (R.T.); sarah.alkandari@dasmaninstitute.org (S.A.-K.); text.jacob@dasmaninstitute.org (T.J.); 2 Kuwait Ministry of Health, Immunology Unit, Mubarak Al Kabeer Hospital, Kuwait City 30000, Kuwait; 3 Animal & Imaging Core Facility, Dasman Diabetes Institute, Kuwait City 15462, Kuwait; Sardar.Sindhu@dasmaninstitute.org (S.S.); ashraf.madhoun@dasmaninstitute.org (A.A.M.); 4 Genetics and Bioinformatics Department, Dasman Diabetes Institute, Kuwait City 15462, Kuwait; fahd.almulla@dasmaninstitute.org; 5 School of Medicine, Royal College of Surgeons, D02 YN77 Dublin 2, Ireland; wahabalghaith@gmail.com

**Keywords:** repetitive intermittent hyperglycemia, glucose fluctuations, IRF5, TLR4, macrophages, type-2 diabetes, metabolic inflammation

## Abstract

Repetitive intermittent hyperglycemia (RIH) is an independent risk factor for complications associated with type-2 diabetes (T2D). Glucose fluctuations commonly occur in T2D patients with poor glycemic control or following intensive therapy. Reducing blood glucose as well as glucose fluctuations is critical to the control of T2D and its macro-/microvascular complications. The interferon regulatory factor (IRF)-5 located downstream of the nutrient sensor toll-like receptor (TLR)-4, is emerging as a key metabolic regulator. It remains unclear how glucose fluctuations may alter the IRF5/TLR4 expression and inflammatory responses in monocytes/macrophages. To investigate this, first, we determined IRF5 gene expression by real-time qRT-PCR in the white adipose tissue samples from 39 T2D and 48 nondiabetic individuals. Next, we cultured THP-1 macrophages in hypo- and hyperglycemic conditions and compared, at the protein and transcription levels, the expressions of IRF5, TLR4, and M1/M2 polarization profile and inflammatory markers against control (normoglycemia). Protein expression was assessed using flow cytometry, ELISA, Western blotting, and/or confocal microscopy. IRF5 silencing was achieved by small interfering RNA (siRNA) transfection. The data show that adipose IRF5 gene expression was higher in T2D than nondiabetic counterparts (*p* = 0.006), which correlated with glycated hemoglobin (HbA1c) (r = 0.47/*p* < 0.001), homeostatic model assessment of insulin resistance (HOMA-IR) (r = 0.23/*p* = 0.03), tumor necrosis factor (TNF)-α (r = 0.56/*p* < 0.0001), interleukin (IL)-1β (r = 0.40/*p* = 0.0009), and C-C motif chemokine receptor (CCR)-2 (r = 0.49/*p* < 0.001) expression. IRF5 expression in macrophages was induced/upregulated (*p* < 0.05) by hypoglycemia (3 mM/L), persistent hyperglycemia (15 mM/L–25 mM/L), and RIH/glucose fluctuations (3–15 mM/L) as compared to normoglycemia (5 mM/L). RIH/glucose fluctuations also induced M1 polarization and an inflammatory profile (CD11c, IL-1β, TNF-α, IL-6, and monocyte chemoattractant protein (MCP)-1) in macrophages. RIH/glucose fluctuations also drove the expression of matrix metalloproteinase (MMP)-9 (*p* < 0.001), which is a known marker for cardiovascular complication in T2D patients. Notably, all these changes were counteracted by IRF5 silencing in macrophages. In conclusion, RIH/glucose fluctuations promote the M1 polarization and inflammatory responses in macrophages via the mechanism involving TLR4-IRF5 pathway, which may have significance for metabolic inflammation.

## 1. Introduction

Intermittent hyperglycemia is considered an independent risk factor for the occurrence of chronic diabetic complications [1]. In clinical practice, glucose fluctuations often occur in patients with poor glycemic control. Intermittent hyperglycemic episodes are also not uncommon soon after induced stress conditions, such as acute infections [2], sepsis [3], and trauma [4], all of which may impair the recovery process of critical conditions and may increase the rate of hospital mortality in critically ill patients [3,5]. Even, the daily glucose fluctuations affected vessel healing in coronary artery disease patients who received everolimus-eluting stent implantations [6].

Oxidative stress, inflammation, and apoptosis of cardiac endothelial cells are the potential mechanisms wielding the impact of intermittent hyperglycemia on vascular endothelial dysfunction and enhancing risks for macrovascular and microvascular complications in patients with type-2 diabetes (T2D) [7,8]. Intermittent hyperglycemia in the rat model induced increased monocyte adhesion to the endothelium compared to that observed in animals with stable glucose, mainly due to overexpression of adhesion molecules such as intercellular adhesion molecule (ICAM)-1 [9,10]. These studies recommended that not only lowering blood glucose but also reducing glucose fluctuations should be considered as an important target for controlling diabetes and related macro- or microvascular complications.

The interferon regulatory factor (IRF) family plays a significant role in the regulation and differentiation of immune cells and adipocytes through different pattern recognition receptors (PRR) including toll-like receptors (TLRs) as well as in the expression of type I interferons (INF α/β) [11,12]. IRFs are also known as the key regulators of metabolic inflammation, and their expression levels are expected to influence the immunobiological consequences in patients with obesity and T2D [13,14,15]. The role of IRF5 in proinflammatory M1 macrophage polarization has been well documented [16,17]. Macrophages are regarded as the central mediators of adipose inflammation and as regulators of adipose tissue homeostasis and insulin sensitivity, involving activation of innate immune receptors, transcription factors, and a shift in intracellular metabolism [18]. IRF5 has turned out to be a direct target gene of P53 that is upregulated in response to DNA damage [19,20]. IRF5 gene expression has also been shown to be induced by signaling through TLRs [21]. Recently, we reported significant correlations between the increased adipose IRF5 expression and the body mass index, inflammatory markers, and hyperglycemia in nondiabetic overweight/obese individuals [13]. As opposed to several studies investigating the impact of persistent hyperglycemia on monocyte/macrophage responses, still little is known about the effect of repetitive intermittent hyperglycemia (RIH) on monocytic cells and macrophage responses. Herein, we present data showing the induction of inflammatory markers and cytokines/chemokine in response to RIH in human monocytic cells and primary macrophages via an IRF5-dependent mechanism.

## 2. Materials and Methods

### 2.1. Study Population and Anthropometric Measurements

A total of 87 (48 nondiabetic and 39 T2D) individuals were recruited in the study. All participants gave written informed consent, and the study was approved (Protocol #: RA-2010-003) by ethics committee of Dasman Diabetes Institute, Kuwait, in accordance with the World Medical Association (WMA) Declaration of Helsinki—Ethical Principles for Medical Research Involving Human Subjects (Updated: 64th WMA General Assembly, Fortaleza, Brazil, October 2013). Anthropometric measurements were obtained using calibrated portable electronic weighing scales and portable inflexible height measuring bars; the waist circumference was measured using constant tension tape. The body composition was measured using IOI353 Body Composition Analyzer (Jawon Medical, South Korea). The body mass index (BMI) was calculated using the standard formula: BMI = body weight (kg)/height (m^2^). The characteristics of the participants are summarized in Supplementary Table S1.

### 2.2. Clinical Analyses

Peripheral blood was collected from overnight-fasting individuals, and samples were analyzed for fasting blood glucose (FBG), lipid profile, and glycated hemoglobin (HbA1c). Glucose and lipid profiles (plasma triglycerides, high-density lipoprotein (HDL), and cholesterol) were measured using Siemens Dimension RXL chemistry analyzer (Diamond Diagnostics Holliston, MA, USA). Glycated hemoglobin (HbA1c) was measured using Variant device (BioRad, Hercules, CA, USA). Homeostatic Model Assessment of Insulin Resistance (HOMA-IR), a measure of insulin resistance, was calculated from basal (fasting) glucose and insulin concentrations using the following formula: HOMA-IR = fasting insulin (μU/L) × fasting glucose (nM/L)/22.5. Clinical data of the participants are summarized in Supplementary Table S2.

### 2.3. Cell Cultures

Human monocytic THP-1 cells were purchased from the American Type Culture Collection (ATCC) and grown in Dulbecco’s modified Eagle’s medium (DMEM) culture medium (Gibco, Life Technologies, Grand Island, USA) supplemented with 10% fetal bovine serum (Gibco, Life Technologies, Grand Island, NY, USA), 5 mM D-glucose (Sigma), 2 mM glutamine (Gibco, Invitrogen, Grand Island, NY, USA), 1 mM sodium pyruvate, 10 mM HEPES, 100 μg/mL normocin, 50 U/ mL penicillin, and 50 μg/mL streptomycin (P/S; Gibco, Invitrogen, Grand Island, NY, USA) by incubation at 37 °C (with humidity) under 5% CO_2_ concentration [22,23,24]. Cells were differentiated into macrophages using phorbol 12-myristate 13-acetate (PMA) for 3 days, followed by overnight conditioning in 5 mM D-glucose serum-free DMEM medium before cell treatments. To investigate the effect of RIH (i.e., glucose fluctuations), THP-1 macrophages were cultured in serum-free DMEM medium supplemented with varying glucose concentrations as follows: (i) hypoglycemia (3 mM/L glucose); (ii) normoglycemia (5 mM/L glucose); (iii) persistent medium hyperglycemia (15 mM/L glucose); (iv) persistent strong hyperglycemia (25 mM/L glucose); and (v) RIH or glucose fluctuations (alternating between 3 mM/L and 15 mM/L glucose, every 12 h) to mimic the RIH conditions relevant to T2D patients. All cultures were incubated at 37 °C (5% CO_2_ with humidity) for 3 days with media changes at every 12 h. For osmolar control, macrophages were cultured in 25 mM/L mannitol under similar incubation and maintenance conditions.

### 2.4. Real-Time qRT-PCR

Total RNA was extracted using RNeasy Mini Kit (Qiagen, Valencia. CA, USA) per the manufacturer’s instructions. The cDNA was synthesized using 1 μg of total RNA using high capacity cDNA reverse transcription kit (Applied Biosystems, Foster city, CA, USA) and following the manufacturer’s instructions [25]. Real-time qPCR was performed on 7500 Fast Real-Time PCR System (Applied Biosystems, Foster City, CA, USA) using TaqMan^®^ Gene Expression Master Mix (Applied Biosystems, Foster city). Each reaction contained 50 ng cDNA that was amplified with Inventoried TaqMan Gene Expression Assay products (IRF5 assay ID: Hs00158114_m1; ITGAX/CD11c assay ID: Hs00174217_m1; interleukin (IL)-1β assay ID: Hs01555410_m1; tumor necrosis factor (TNF)-α assay ID: Hs01113624_g1; IL-5 assay ID: Hs01548712_g1; IL-10 assay ID: Hs00961622_m1; and glyceraldehyde 3-phosphate dehydrogenase (GAPDH) assay ID: Hs03929097_g1). The threshold cycle (Ct) values were normalized to GAPDH, and the amounts of targets’ mRNA relative to control were calculated using 2^−ΔΔCt^ method [26,27]. Relative mRNA expression was expressed as fold change over the average of GAPDH expressions. The expression level in the control treatment was taken as 1.

### 2.5. Flow Cytometry

Cultured macrophages were treated as mentioned above. For analyzing surface markers expression, cultures were incubated with PBS-EDTA for 20 min to detach the cells. Cells were resuspended in fluorescence-activated cell sorting (FACS) staining buffer (BD Biosciences) and blocked with human IgG (Sigma; 20 μg) for 30 min on ice. Cells were washed and resuspended in 100 μL of FACS buffer and incubated with anti-CD11c (S_HCL-3)-PE (cat# 347637; BD Biosciences) or anti-CD11c PE-Cy7 (cat # 117317; BD Biosciences ), anti-CD206-APC (cat# 561763; BD Biosciences), anti-CD163-PE (cat# 556018; BD Biosciences), anti-CD14-APC (cat# 555399; BD Biosciences), and anti-TLR-4 FITC (cat#ab8378; Abcam) on wet ice for 30 min, with gentle mixing every 10 min. Cells were washed three times with FACS buffer and fixed by resuspending in 2% paraformaldehyde solution [28]. Cells were centrifuged and resuspended in FACS buffer for FACS analysis (FACSCanto II; BD Bioscience, San Jose, USA), and data were analyzed using BD FACSDiva^TM^ Software 8 (BD Biosciences, San Jose, USA).

For analyzing intracellular markers expression, cells were seeded in 24-well plates and treated as mentioned above. Cells were detached using PBS-EDTA for 20 min, followed by 3 washes with PBS. Cells were incubated with fixation/permeabilization buffer (cat# 554714, eBioscience, San Diego, CA, USA) for 20 min on wet ice, followed by 3 washes with Perm/Wash™ buffer and incubated with anti-IL-1β-PE (cat# 340516; BD Biosciences), anti-IL-6-PE (cat# 551850; BD Biosciences), anti-IL-4-Alexa Fluor^®^ 647 (cat#564084; BD Biosciences), or anti-IL-10-FITC (cat#11710182; BD Biosciences) for 30 min. Cells were washed thrice and resuspended in PBS supplemented with 2% FBS for FACS and data analysis as described before.

### 2.6. ELISA

Macrophages were cultured and treated as mentioned above, and supernatants were collected after the last 12 h incubation. Secreted IL-1β and MCP-1 proteins were measured in the supernatants using sandwich ELISA, following the manufacturers’ instructions (R&D systems, Minneapolis, MN, USA) [29].

### 2.7. Small Interfering RNA (siRNA) Transfections

Monocytes were washed and resuspended in 100 μL of nucleofector solution (Amaxa Nucleofector Kit V, Lonza, Germany) and transfected separately with IRF5-siRNA (30 nM; OriGene Technologies, Inc. Rockville, MD, USA) or scrambled-siRNA (negative control) (30 nM; OriGene Technologies, Inc. MD, USA) and pmaxGFP (0.5 μg; Amaxa Nucleofector Kit V, Lonza, Köln, Germany). All transfections were performed with Amaxa Cell Line Nucleofector Kit V (Lonza, Germany) and by using Amaxa Electroporation System (Amaxa Inc; Cologne, Germany) following the protocol as described elsewhere [30]. After 36 h of transfection, cells were plated for treatments as mentioned before. In IRF5 knockdown experiments, IRF5 protein expression was assessed by using standard protocol for Western blot.

### 2.8. Confocal Microscopy

For detecting protein expression by confocal microscopy, THP-1 cells were seeded on a coverslip and allowed to settle by incubation for 4 h. Cells were then transformed to macrophages and treated with different glucose concentrations as described before. Later, cells were treated with 4% paraformaldehyde for 20 min and stained as follows. Briefly, the samples were treated overnight at room temperature with rabbit polyclonal anti-human primary antibodies against MMP-9 (diluted 1:100, Abcam^®^ab76003). After 3 washes with PBS-Tween, samples were incubated for 1 h with secondary antibody Alexa Fluor^®^488-conjugated Ab (diluted 1:200, Abcam^®^ab150077), followed by three more washes. Cells were counterstained and mounted over slides using mountant containing DAPI (Vectashield, Vectorlab, H1500). Confocal images were obtained by using inverted Zeiss LSM710 spectral confocal microscope (Carl Zeiss, Gottingen, Germany) and EC Plan-Neofluar 40×/1.30 oil DIC M27 objective lens. After exciting samples with a 543 nm HeNe laser and 405 nm line of an argon ion laser, optimized emission detection bandwidths were configured by using Zeiss Zen 2010 control software [31]. All samples were analyzed using the same parameters, and the resulting color markup of analysis was confirmed for each sample.

### 2.9. Statistical Analysis

Data are shown as mean ± SEM values, and statistical analysis was performed using Prism 8.3.1 software (GraphPad Inc., San Diego, CA, USA). Group mean were compared using unpaired Student’s *t*-test, and the strength of the association between two variables was measured by Pearson’s correlation coefficient (r). All *p*-values ≤ 0.05 were considered as statistically significant.

## 3. Results

### 3.1. Demographic and Clinical Characteristics of the Study Population

The demographic and clinical characteristics of the study participants are summarized in Supplementary Tables S1 and S2, respectively. There were no significant differences between nondiabetic and diabetic groups regarding age, body weight, BMI, or fat percentage. As anticipated and compared with nondiabetics, T2D patients not only reflected insulin resistance as indicated by their elevated fasting glucose (*p* < 0.0001), insulin (*p* = 0.0006), and homeostatic model assessment of insulin resistance (HOMA-IR) (*p* = 0.042 but also had significantly high levels of triglycerides (*p* < 0.0001), a common marker of patients with uncontrolled T2D [32].

### 3.2. Elevated Adipose IRF5 Gene Expression in Diabetic Patients as Well as IRF5 Induction in Macrophages by Repetitive Intermittent Hyperglycemia (RIH)

**Figure 1:**
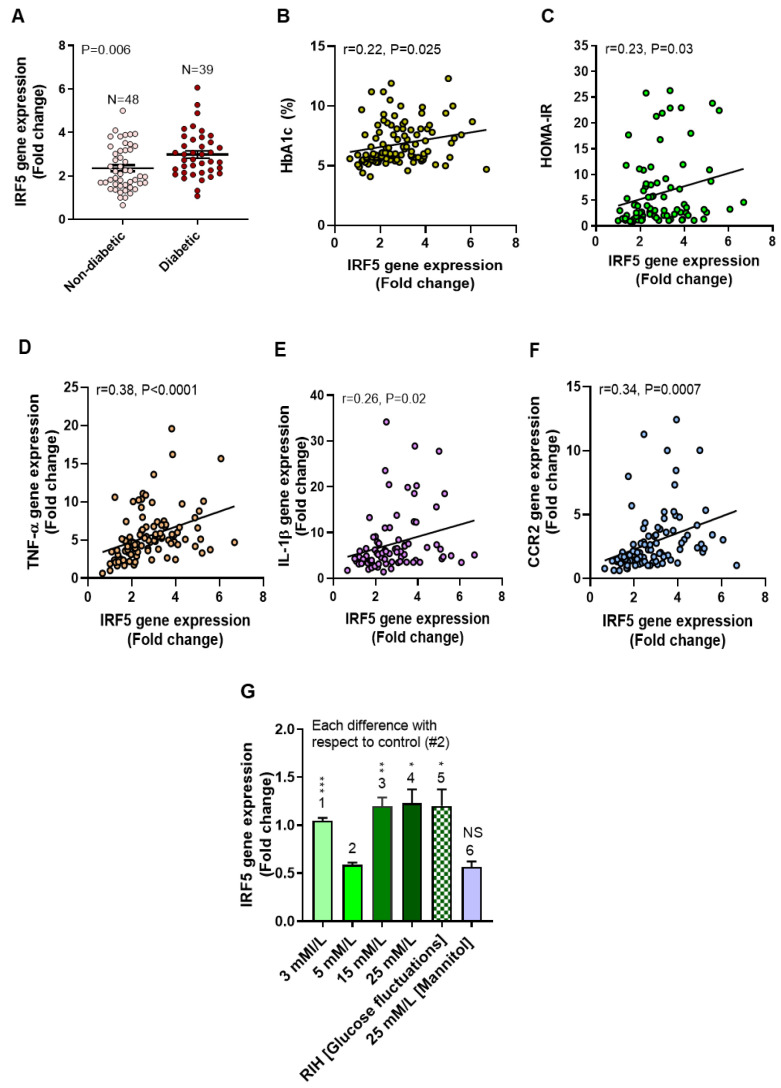
Adipose IRF5 gene expression is upregulated in type-2 diabetic patients as well as in macrophages cultured under low, high, and repetitive intermittent hyperglycemia (RIH). Adipose tissue samples from nondiabetic (N = 48) and diabetic (N = 39) individuals were analyzed for transcripts of interferon regulatory factor (IRF5), tumor necrosis factor (TNF)-α, interleukin (IL)-1β, and C-C motif chemokine receptor (CCR)-2 using real-time qRT-PCR as described in the Materials and Methods section. Glycated hemoglobin (HbA1c) levels were determent by biochemical analysis of whole blood samples. In addition, THP-1 monocytic cells were differentiated into macrophages and cultured for 3 days under conditions of hypoglycemia (3 mM/L), normoglycemia (5 mM/L), persistent medium and strong hyperglycemia (15 mM/L and 25 mM/L, respectively), and RIH/glucose fluctuations (3–15 mM/L). Macrophages were cultured with mannitol (25 mM/L) for osmolar control. (**A**) Adipose IFR5 mRNA expression was found to be higher in diabetic than nondiabetic individuals (*p* = 0.006). The increased adipose IRF5 expression correlated positively with (**B**) HbA1c (r = 0.47, *p* < 0.0001), (**C**) Homeostatic Model Assessment of Insulin Resistance (HOMA-IR) (r = 0.23, *p* = 0.03), (**D**) TNF-α (r = 0.56, *p* < 0.0001), (**E**) IL-1β (r = 0.40, *p* = 0.0009), and (**F**) CCR2 (r = 0.49, *p* < 0.0001). (**G**) IRF5 gene expression was upregulated in THP-1 macrophages following exposure to hypoglycemia, persistent medium and strong hyperglycemia, and RIH/glucose fluctuations as compared to control (normoglycemia). All data are expressed as mean ± SEM values. * *p* ≤ 0.05, ** *p* ≤ 0.01, *** *p* ≤ 0.001, and NS: nonsignificant.

Previously, we showed that adipose IRF5 expression was higher in obese individuals compared to lean and overweight, which was found to associate with several metabolic and inflammatory markers [13,14]. We further showed by confocal microscopy that the adipose tissue macrophages and not the adipocytes expressed IRF5 transcripts [13,14]. In line with these observations, first, we show that, in our T2D patient cohort, adipose IRF5 gene expression was significantly higher than their nondiabetic counterparts (*p* = 0.006, Figure 1A). This upregulation of IRF5 in T2D patients associated positively with the hyperglycemia-related clinical markers HbA1c (r = 0.47, *p* < 0.0001, Figure 1B) and HOMA-IR (r = 0.23, *p* = 0.03, Figure 1C) as well as with critical inflammatory markers including TNF-α (r = 0.56, *p* < 0.0001, Figure 1D), IL-1β (r = 0.40, *p* = 0.0009, Figure 1E), and C-C motif chemokine receptor (CCR)-2 (r = 0.49, *p* < 0.0001, Figure 1F).

We next asked whether the glycemic modulations could affect IRF5 expression in macrophages. Notably, both hyperglycemia [33,34] and hypoglycemia [35,36] have been implicated in the progression of T2D-associated pathologies. Therefore, to test the effects of both high and low glucose concentrations on IRF5 expression, we cultured the THP-1-derived macrophages under five different glycemic conditions as described in the Materials and Methods section. As shown in Figure 1G, IRF5 gene expression was significantly upregulated in macrophages cultured under hypoglycemia (*p* = 0.0003), persistent medium hyperglycemia (*p* = 0.003), and persistent strong hyperglycemia (*p* = 0.01) as compared to normoglycemic control. Interestingly, IRF5 expression was also upregulated in macrophages that were cultured under the RIH or glucose fluctuations as compared to normoglycemic control (*p* = 0.02), which was comparable (*p* > 0.05) to IRF5 expression under the persistent hyperglycemia at 15 mM/L and 25 mM/L concentrations. However, IRF5 expression remained unaltered in macrophages that were cultured in 25 mM/L mannitol, comparable with IRF5 expression in normoglycemia (*p* = 0.73). These data indicate that IRF5 gene expression in macrophages was upregulated by persistent or intermittent hyperglycemia as well as by hypoglycemia, whereas these changes were not related to the osmolar factor.

### 3.3. IRF5 Upregulation Is Paralleled by Expression of M1 Macrophage Polarization Markers and Pro-Inflammatory Cytokines

**Figure 2:**
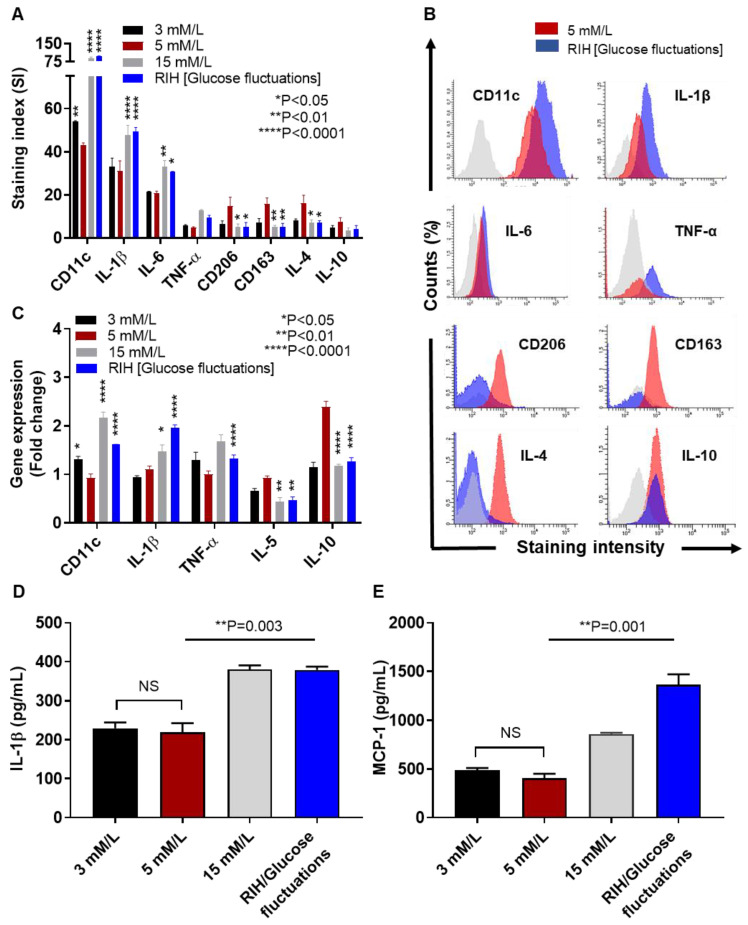
IRF5 upregulation is accompanied by increased M1 macrophage polarization markers and pro-inflammatory cytokines expression. THP-1 transformed macrophages were cultured for 3 days under conditions of hypoglycemia (3 mM/L), normoglycemia (5 mM/L), persistent medium hyperglycemia (15 mM/L), and RIH/glucose fluctuations (3–15 mM/L). Cells were harvested and stained with fluorescent antibodies against M1/M2 macrophage markers and selected pro-inflammatory and anti-inflammatory cytokines as described in the Materials and Methods section. IL-1β and monocyte chemoattractant protein (MCP)-1 levels in culture supernatants were measured by ELISA. Cells were also harvested for measuring gene expression of M1/M2 polarization and inflammatory markers using real-time qRT-PCR. (**A**) Representative flow cytometry data from three independent determinations with similar results are presented as bar graphs of mean staining index (SI). (**B**) Representative flow cytometry data from three independent determinations with similar results are presented as histograms. (**C**) Representative qRT-PCR data from three independent determinations with similar results are presented as bar graphs showing selected gene expression as fold change over control gene expression taken as 1. (**D**,**E**) Representative ELISA data from three independent determinations with similar results are presented as bar graphs showing secreted protein levels of IL-1β and MCP-1, respectively, in macrophage culture supernatants. All data are expressed as mean ± SEM values. * *p* ≤ 0.05, ** *p* ≤ 0.01, **** *p* ≤ 0.0001, and NS: nonsignificant.

Increased IRF5 expression is related to macrophage polarization shift from an anti-inflammatory M2 to a pro-inflammatory M1 phenotype. We, therefore, asked if culturing under (i) hypoglycemia (3 mM/L), (ii) persistent hyperglycemia (15 mM/L), and (iii) RIH or glucose fluctuations (3–15 mM/L) could affect the M1/M2 markers expression in macrophages. To this end, flow cytometry data show a significant increase in the M1 markers (CD11c, IL-1β, and IL-6) and decrease in the M2 markers (CD206, CD163, and IL-4) under persistent hyperglycemia as well as RIH/glucose fluctuations as compared to normoglycemic control (Figure 2A); however, under hypoglycemia, only the CD11c expression was found to be significantly elevated compared to normoglycemic controls (Figure 2A). Selected histogram images of the changes in M1 markers (CD11c, IL-1β, IL-6, and TNF-α) and M2 markers (CD206, CD163, IL-4, and IL-10) are compared in RIH/fluctuating glycemia as compared to normoglycemia (Figure 2B). As expected, these changes in protein expression were found to be concordant with the transcriptional modulations in cultured macrophages which displayed upregulated gene expression of M1 (CD11c, TNF-α, and IL-1β) and downmodulated M2 (IL-5) markers under constant or fluctuating hyperglycemia (Figure 2C). Together, these data imply that M1 polarization is favored as macrophages are cultured under RIH or fluctuating hyperglycemia, with a similar macrophage markers expression when macrophages are cultured under constant hyperglycemia.

In addition to the phenotypic changes, we also evaluated the bio-functional response in hyperglycemia-exposed macrophages by measuring secretion of critical proinflammatory proteins, such as IL-1β and MCP-1 (CCL2). In line with our previous observations, IL-1β (Figure 2D) and MCP-1 (Figure 2E) levels were significantly elevated in supernatants from macrophages cultured under the constant hyperglycemia (IL-1β/*p* = 0.003; MCP-1/*p* = 0.001) or fluctuating hyperglycemia (IL-1β/*p* = 0.003; MCP-1/*p* = 0.001) as compared to respective normoglycemic controls. Interestingly, IL-1β and MCP-1 expression in macrophages cultured under hypoglycemia compared with their expression in the normoglycemic controls, respectively (*p* > 0.05).

### 3.4. IRF5 Suppression Prevents the Expression of M1 Markers and Inflammatory Cytokines/Chemokine in Macrophages Cultured under RIH/Glucose Fluctuations

**Figure 3:**
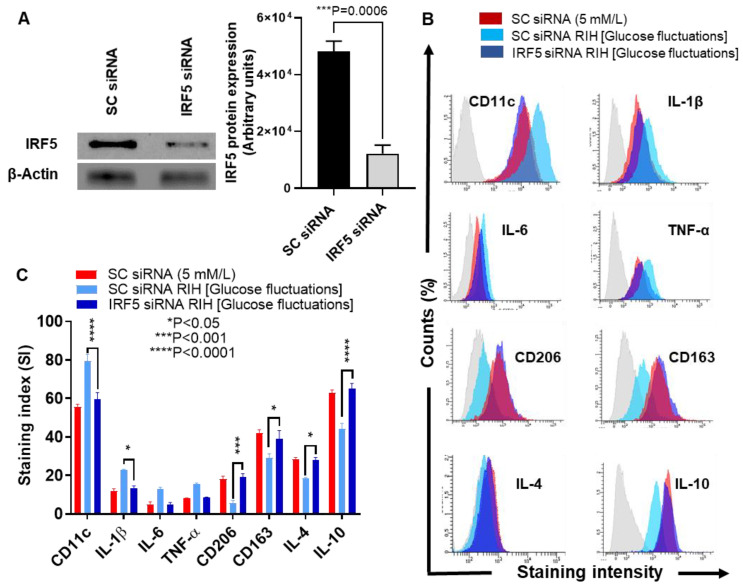

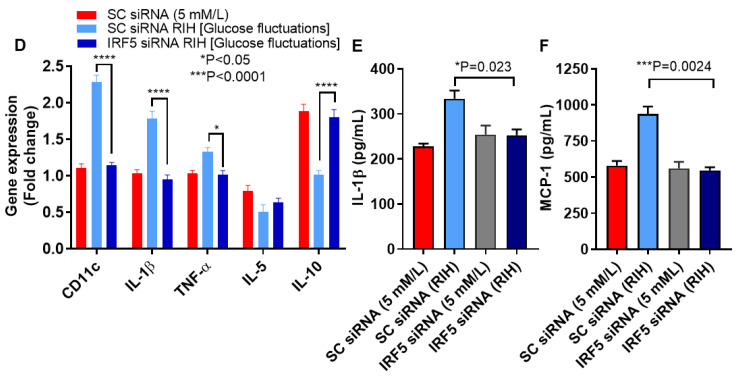
IRF5 silencing prevents the expression of M1 markers and inflammatory cytokines/chemokine in THP-1 macrophages cultured under repetitive intermittent hyperglycemia (RIH). THP-1 monocytes were transfected with scrambled siRNA (mock/negative control) or IRF5 siRNA and incubated for 36 h for transformation into macrophages following standard protocol. THP-1 macrophages were then cultured for 36 h under conditions of normoglycemia (5 mM/L) and RIH/glucose fluctuations (3–15 mM/L). Cells were harvested and stained with fluorescent antibodies against M1/M2 macrophage markers and selected pro-inflammatory and anti-inflammatory cytokines as described in the Materials and Methods section. IRF5 protein expression in transfected cells was measured by Western blot. IL-1β and MCP-1 levels in culture supernatants were measured by ELISA. Cells were also harvested for measuring gene expression of M1/M2 polarization and inflammatory markers using real-time qRT-PCR as described in the Materials and Methods section. (**A**) Representative data from three independent determinations with similar results show the diminished IRF5 protein expression in transfected cells compared with the mock cells (*p* = 0.0006). (**B**) Representative flow cytometry data from three independent determinations with similar results are presented as histograms. (**C**) Representative flow cytometry data from three independent determinations with similar results are presented as bar graphs of protein expression, shown as mean staining index (SI). (**D**) Representative qRT-PCR data from three independent determinations with similar results are presented as bar graphs showing selected gene expression as fold change over control gene expression taken as 1. (**E**,**F**) Representative ELISA data from three independent determinations with similar results are presented as bar graphs showing secreted protein levels of IL-1β and MCP-1, respectively, in macrophage culture supernatants. All data are expressed as mean ± SEM values. * *p* ≤ 0.05, *** *p* ≤ 0.001, and **** *p* ≤ 0.0001.

We next asked if M1 polarization and inflammatory cytokines/chemokine expression could be prevented by genetic ablation of IRF5 in macrophages cultured under RIH or glucose fluctuations. To this end, we transfected macrophages with IRF5-specific siRNA and verified downmodulation of IRF5 protein compared to controls transfected with scrambled siRNA (*p* = 0.0006, Figure 3A). In this regard, our data show that IRF5-depleted cells had lower protein expression of M1 inflammatory markers including CD11c (*p* < 0.0001) and IL-1β (*p* = 0.0186) and, conversely, higher protein expression of anti-inflammatory M2 markers including CD206 (*p* = 0.0003), CD163 (*p* = 0.0151), IL-4 (*p* = 0.0177), and IL-10 (*p* < 0.0001) (Figure 3B,C). As expected, IRF5-defective macrophages displayed the reduced gene expression of a number of inflammatory genes including CD11c (*p* < 0.0001), IL-1β (*p* < 0.0001), and TNF-α (*p* = 0.0371). On the other hand, these cells showed elevated gene expression of anti-inflammatory cytokine IL-10 (*p* < 0.0001) (Figure 3D). Regarding the functional activity, macrophages transfected with IRF5-specific siRNA displayed the attenuated expression of signature inflammatory proteins including IL-1β (*p* = 0.023) (Figure 3E) and MCP-1 (*p* = 0.0024) (Figure 3F). These data collectively suggest that IRF5 deficiency in macrophages inhibits the M1 macrophage polarization and expression of inflammatory proteins in response to RIH/glucose fluctuations.

### 3.5. RIH Promotes the Expression of TLR4 on Monocytes/Macrophages

**Figure 4:**
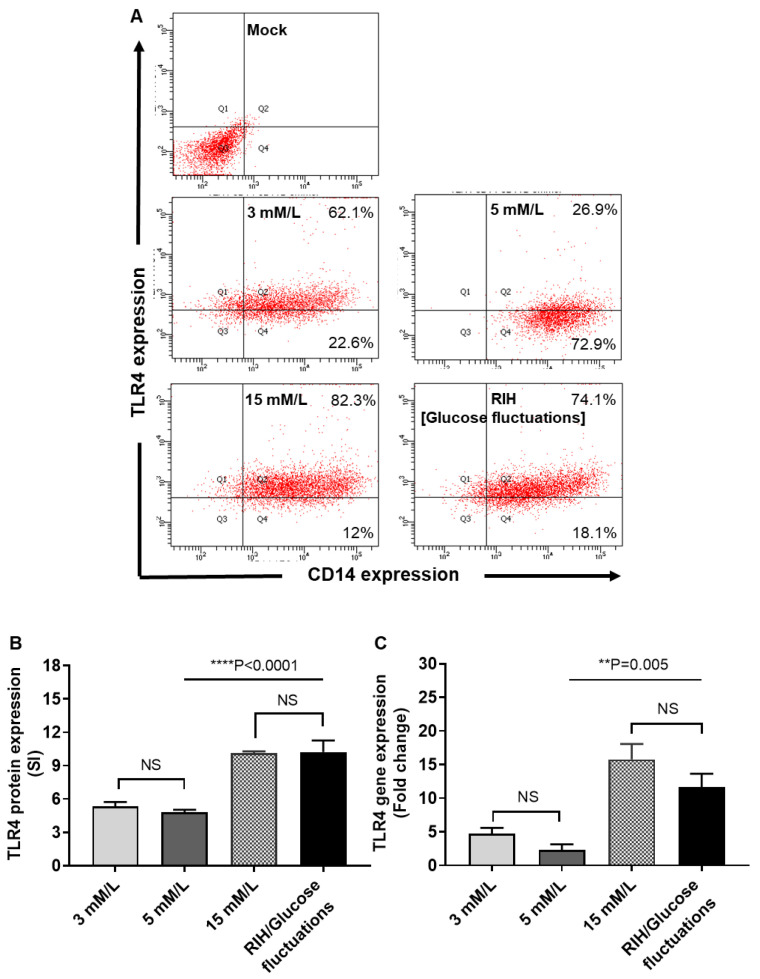
Repetitive intermittent hyperglycemia (RIH) promotes the expression of TLR4 on THP-1 macrophages. THP-1 transformed macrophages were cultured for 3 days under conditions of hypoglycemia (3 mM/L), normoglycemia (5 mM/L), persistent medium hyperglycemia (15 mM/L), and RIH/glucose fluctuations (3–15 mM/L). Cells were harvested and stained with fluorescent antibodies against CD14 and TLR4 along with isotype matched controls. TLR4 gene expression were measured using real-time qRT-PCR as described in the Materials and Methods section. (**A**) Representative flow cytometry data from three independent determinations with similar results are presented as dot blot for TLR4 vs. CD14 expression in macrophages. (**B**) Representative flow cytometry data from three independent determinations with similar results are presented as bar graph of mean staining index (SI). (**C**) Representative qRT-PCR data from three independent determinations with similar results are presented as bar graphs showing TLR4 gene expression as fold change over control gene expression taken as 1. All data are expressed as mean ± SEM values. ** *p* ≤ 0.01, **** *p* ≤ 0.0001, and NS: nonsignificant.

Given that TLR4 and IRF5 play a key role in metabolic inflammation and that our data showed that IRF5 expression was upregulated by glucose fluctuations, we asked whether RIH altered the TLR4 expression as well. There is a paucity of data examining the TLR4 expression changes in monocytes/macrophages following exposure to glycemic alterations. Herein, we determined TLR4 protein and mRNA expression in macrophages cultured under hypo- and hyperglycemia (RIH). To this end, our data show that RIH or glucose fluctuations promoted the TLR4 protein (*p* < 0001, Figure 4A,B) and gene expression (*p* = 0.005, Figure 4C) in macrophages, which differed non-significantly from TLR4 expression in macrophages cultured in persistent hyperglycemia (15 mM/L) (*p* > 0.05). Also, TLR4 changes under hypoglycemia were comparable to those of control (normoglycemia) (*p* > 0.05).

### 3.6. RIH Induces the Expression of Matrix Metalloproteinase (MMP)-9, Enhancing the Risk for Diabetes-Related Pathologies in a Clinical Setting

**Figure 5:**
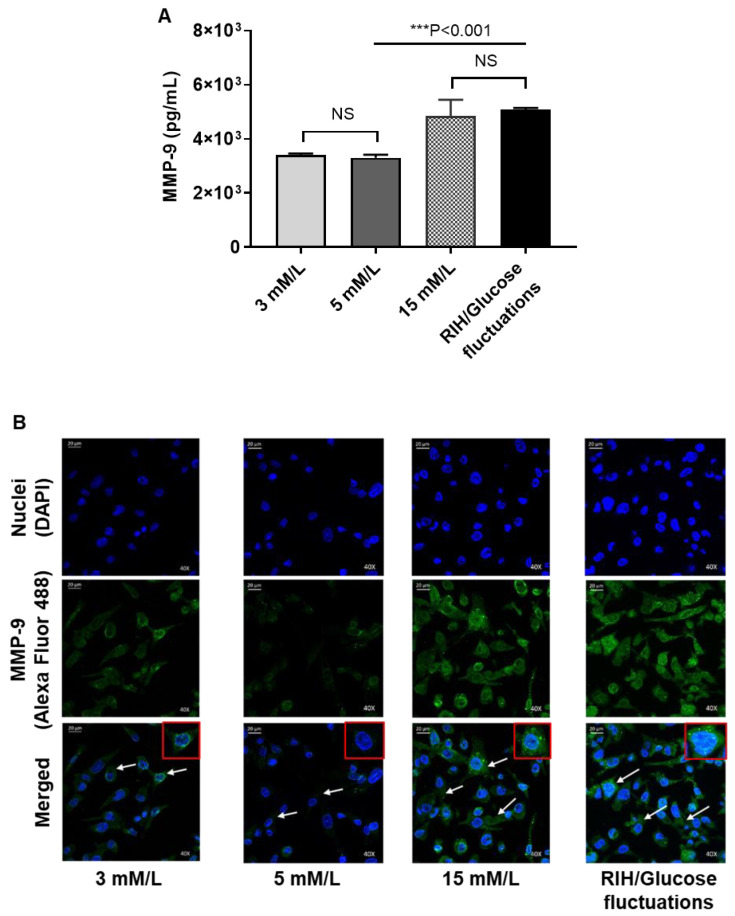

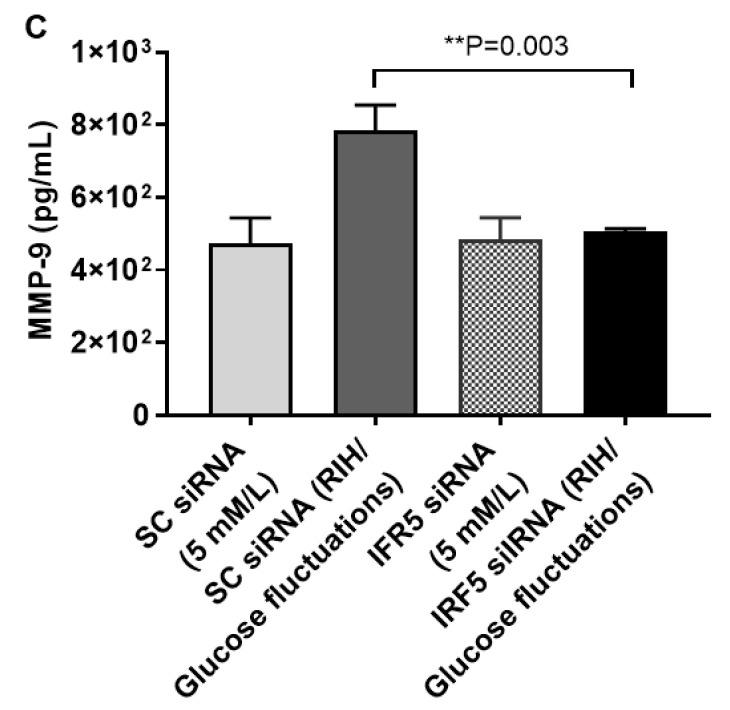
Repetitive intermittent hyperglycemia (RIH) induces IRF5-dependent expression of matrix metalloproteinase (MMP)-9 in THP-1 macrophages. THP-1-derived macrophages grown on coverslips were cultured for 3 days under conditions of hypoglycemia (3 mM/L), normoglycemia (5 mM/L), persistent medium hyperglycemia (15 mM/L), and RIH/glucose fluctuations (3–15 mM/L). MMP-9 intracellular expression and MMP-9 secreted protein in culture supernatants were determined by confocal microscopy and ELISA, respectively, as described in the Materials and Methods section. In IRF5 silencing assays, THP-1 monocytes were transfected with scrambled siRNA (mock/negative control) or IRF5 siRNA and incubated for 36h. Later, THP-1 transformed macrophages were cultured for 36 h under conditions of normoglycemia (5 mM/L) and RIH/glucose fluctuations (3–15 mM/L), and supernatants were collected for measuring MMP-1 secreted protein expression by ELISA. (**A**) Representative ELISA data from three independent determinations with similar results are presented as bar graph of MMP-9 secreted protein expression (pg/mL) in culture supernatants. (**B**) Representative images from five independent determinations with similar results, showing MMP-9 protein expression in macrophages cultured under various glucose concentration in the medium. Images are shown at 40× magnification; Scale bar = 20 µM. (**C**) Representative ELISA data from three independent determinations with similar results are presented as bar graph showing MMP-9 secreted protein expression (pg/mL) in supernatants of macrophages cultured under normoglycemia and RIH/glucose fluctuations, with or without IRF5 silencing. All data are expressed as mean ± SEM values. ** *p* ≤ 0.01, *** *p* ≤ 0.001, and NS: nonsignificant.

M1 to M2 macrophage phenotype transition is known to resolve inflammation and favor tissue repair. We earlier showed that IRF5 silencing in macrophages induced the M2 phenotypic reprogramming marked by ↓CD11c, ↑CD206, ↑CD163, ↑IL4, and ↑IL-10 as well as suppression of the inflammatory cytokines/chemokine (↓IL-1β, ↓TNF-α, and ↓MCP-1). Next, we tested whether the M2 reprogramming and induction of anti-inflammatory response following IRF5 ablation in macrophages could have significance regarding somatic disease pathophysiology, such as cardiovascular disease or cancer metastasis, both of which have increased incidence in obesity/T2D. To this end, first, we show that macrophages exposed to RIH as compared to control (normoglycemia) display the elevated expression of matrix metalloproteinase (MMP)-9 which is an endopeptidase involved in remodeling of extracellular matrix and adhesion proteins (*p* < 0.001, Figure 5A,B). These changes were similar to MMP-9 expression in macrophages that were cultured in persistent hyperglycemia (15 mM/L glucose concentration). However, MMP-9 secretion in macrophages was significantly diminished by IRF5 silencing as compared to controls that were transfected with scrambled siRNA (*p* = 0.003, Figure 5C). Together, these data imply that RIH induces the expression of MMP-9 in macrophages through an IRF5-dependenbt mechanism, downstream of TLR4.

**Figure 6:**
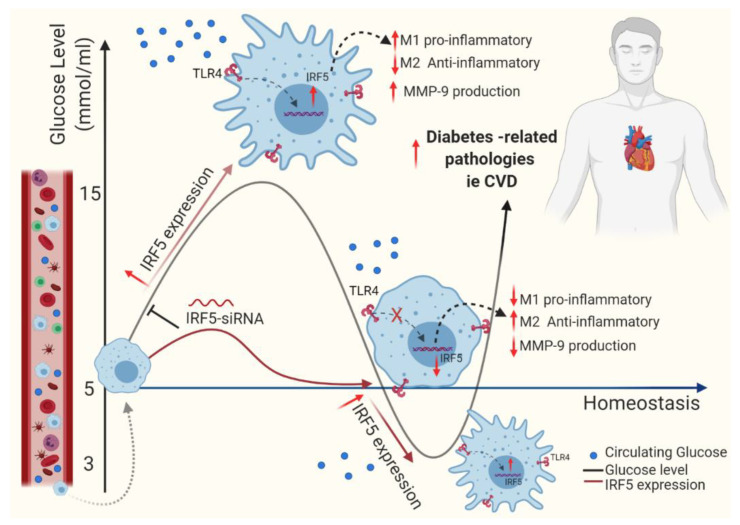
This illustration represents a proposed model of metabolic inflammation in support of the data presented, wherein RIH or glucose fluctuations in vitro upregulate the expression of TLR4-IRF5 together with markers of inflammation and the cardiovascular disease marker MMP-9 in macrophages. These data support the notion that controlling glucose fluctuations and/or IRF5 silencing could be beneficial to alleviate metabolic inflammation.

Overall, the thematic illustration supporting our data is shown below (Figure 6):

## 4. Discussion

In this study, we present the data supporting IRF5-dependent mechanisms by which repetitive intermittent hyperglycemia (RIH) or glucose fluctuations orchestrate the M1 macrophage polarization as well as expression of inflammatory cytokines/chemokine and MMP-9 in the human monocytic THP-1-derived macrophages. First of all, we show that the expression of immune-metabolic transcriptional regulator IRF5 was found to be significantly upregulated in monocytes from diabetic patients compared to nondiabetic individuals and that these changes associated with the expression of hyperglycemia-induced clinical marker HbA1c and several inflammatory markers including TNF-α, IL-1β, and CCR2. These data are corroborated, at least in part, by our previous reports showing enhanced subcutaneous-adipose tissue IRF5 expression in individuals with obesity and/or T2D in association with an inflammatory immune profile in the adipose tissue as well as by the data showing that macrophages and not adipocytes were the IRF5 expressors [13,14]. In line with this, at least in part, an earlier study by Dalmas et al. also reported that IRF5 is the orchestral conductor of macrophage/lymphocyte activation in obesity and is involved in the crosstalk between inflammatory and prodiabetogenic factors. The authors showed that IRF5 deficiency induced a substantial reprogramming of inflammation in the visceral adipose tissue and a tissue remodeling limiting the expansion of intra-abdominal adipocytes with redistribution of lipids to the subcutaneous deposits [37,38].

A positive correlation between HbA1c and IRF5 expression implies that a loss of glycemic control is concomitant with increased IRF5 expression. Further, in this regard, our data show that the increased IRF5 expression also associated positively with HOMA-IR, which is an indicator of the loss of glucose homeostasis and induction of insulin resistance. A positive association between IRF5 expression and HbA1c in our diabetic cohort prompted us to test whether the RIH (glucose fluctuations) could induce or drive IRF5 expression in macrophages. To this end, our data show that glucose fluctuations significantly promote the IRF5 gene expression in THP-1-derived macrophages, with comparable induction by other forms of hyperglycemia, i.e., persistent hyperglycemia involving 15 mM/L and 25 mM/L glucose concentrations. Of note, hyperglycemia has been associated with chronic low-grade inflammation in T2D patients, exacerbating the associated pathologies such as diabetic retinopathy, macular edema, and cardiovascular disease [39,40]. Interestingly, we found that hypoglycemia, whether persistent or repetitive-intermittent, induced the expression of IRF5, nearly 2-fold, in THP-1 macrophages as compared to the normoglycemic control. This observation is novel to our knowledge and points to the plausible deleterious effects of RIH in metabolic inflammation via the induction of IRF5 in macrophages. In T2D patients, chronic low-grade inflammation and oxidative stress over time may impair insulin signaling and may induce a state of insulin resistance, marked by hyperglycemia. On the other hand, hypoglycemia may occur as an “often-neglected” complication of intensive glucose-lowering therapy in T2D patients. Besides, hypoglycemia can also be a complication related to other factors, such as organ failure, pancreatic or endocrine disease, autoimmune disorders, infections, sepsis, toxins, stress, and starvation [41]. Slight fluctuations in blood sugar are considered normal; however, recurrent and significant glycemic modulations in T2D patients are related with serious complications such as myocardial infarction, retinal degenerative changes, loss of vision, neurocognitive dysfunction, aggravation of acute cerebrovascular disease, and poor quality of life [42].

Next, our data show upregulation of the inflammatory M1 markers (CD11c, IL-1β, TNF-α, and IL-6) and downmodulation of the anti-inflammatory M2 markers (CD206, CD163, IL-5, and IL-10) in the macrophages cultured under RIH as compared to normoglycemic controls. In order to further understand the biological significance of this macrophage polarization shift, we measured inflammatory protein secretion by these macrophages and found that macrophages exposed to RIH expressed increased levels of IL-1β and MCP-1. Together, these data imply that glucose fluctuations per se, without additional metabolic factors, could act as a potent trigger for the differentiation and programming of macrophages, favoring an inflammatory M1 phenotype. In line with these observations, Grosick et al. reported that hyperglycemia induced priming effects in THP-1 macrophages, exacerbating the production of proinflammatory cytokines including TNF-α, IL-6, and MCP-1 [43]. Consistent with our data showing metabolic reprogramming effects of hyperglycemia in macrophages, Pavlou et al. also reported that the sustained high glucose exposure could sensitize macrophage responses, leading to enhanced expression of IL-1β and TNF-α but with a reduced phagocytic activity [44]. Interestingly, Nagareddy et al. demonstrated that, in obese mice, IL-1β expression from CD11c^+^ adipose tissue macrophages further promoted adipose inflammation by enhancing bone marrow myelopoiesis and monocytosis [45]. Similarly, another study also reported that proinflammatory CD11c^+^ macrophages associated positively with systemic insulin resistance in obese patients [46]. Our data showing the M1-like polarization effects of hyperglycemia are in agreement with another study reporting the increased CD11c expression and reduced CD206 and IL-10 expression in primary human monocytes/macrophages cultured under high glucose concentration as well as in circulating monocytes from patients with hyperglycemia [47]. The increased MCP-1 secretion by THP-1 macrophages that we observed following exposure to RIH implies that these macrophages could, in principle, influence the migration and infiltration of critical immune effectors, such as monocytes, memory T lymphocytes, and NK cells and could impact the inflammatory responses. Notwithstanding, it has also been pointed out that the possible local pathogenic role of MCP-1 may not be reflected by the increased systemic levels of MCP-1 in obesity or T2D [48]. Indeed, Amano et al. showed that, in the visceral white adipose tissue, MCP-1 could promote macrophage proliferation, independently of its chemokine function (i.e., monocyte chemoattraction) [49]. Interestingly, our data show that, though hypoglycemia could also drive a M1 polarization shift, unlike hyperglycemia-induced programming, these macrophages did not secrete increased levels of IL-1β and MCP-1.

In further elucidating the underlying mechanisms of M1-like macrophage polarization and inflammatory cytokines/chemokine production in response to RIH, we found that hyperglycemia-driven changes were counteracted as the expression of IRF5 was silenced in macrophages. Thus, IRF5-ablated macrophages displayed a M2 phenotype which was marked by a higher expression of anti-inflammatory (CD206, CD163, IL-4, and IL-10) and a lower expression of inflammatory markers (CD11c, IL-1β, and TNF-α). As expected, this reversal of macrophage polarization or reprogramming into a M2-phenotype in the IRF5-deficient macrophages was accompanied by a reduced expression of IL-1β and MCP-1secretroy proteins, implying that IRF5 was essentially required for the induction of M1 polarization and inflammatory changes triggered by glucose fluctuations. IRF5 transcription factor was originally implicated in antiviral response and type-I interferon production. Nonetheless, studies emerging over the last decade support the role of IRF5 as a key regulator of inflammation and it was found to be involved in the pathogenesis of several inflammatory or autoimmune diseases including inflammatory bowel disease, rheumatoid arthritis, and systemic lupus erythematosus [50,51,52,53,54]. The role of IRF5 as a key transcriptional regulator of M1 macrophage polarization and metabolic inflammation is also supported by other studies [18,55]. Consistent with our results supporting IRF5-dependent M1 polarization and inflammatory programming in macrophages, Wei et al. showed that IRF5 deficiency in myeloid cells could prevent the pathogenesis of necrotizing enterocolitis through inhibiting the M1 macrophage polarization [17]. Likewise, Krausgruber et al. also showed that IRF5 was involved in TNF-α secretion by human dendritic cells [56]. Interestingly, in a recently published review, Laviada-Molina et al. argued that overproduction of deleterious cytokines called “cytokine storm” following severe acute respiratory syndrome coronavirus 2 (SARS-CoV-2) infection was related to the increased glucose metabolism and IRF5 triggering, leading to hyperinflammation due to a massive inflammatory gene overexpression, induction of the ER stress, and a dysregulated cytokine profile, with increased risks of vascular hyperpermeability and multiorgan failure [57].

We further show that expression of the innate immune TLR4 receptor was enhanced in THP-1 macrophages exposed to RIH as compared to normoglycemia while it matched with the TLR4 expression induced by persistent hyperglycemia at a glucose concentration of 15 mM/L. This observation is intriguing in the perspective that TLR4 is emerging as a nutrient sensor and that elevated TLR4 expression has been documented in metabolic disorders including obesity and/or T2D. Consistent with our data showing TLR4 upregulation in macrophages in response to RIH, Wang et al. found that hyperglycemia induced the overexpression and activation of TLR4 in endothelial cells [58]. Of note, TLR4 expression is enhanced significantly in adipose tissue macrophages during obesity [59] and TLR4/TLR2 expression was found to be significantly higher in M1 compared to M2 macrophages [60]. Dasu et al. also reported that high glucose induced TLR2 and TLR4 expression in human monocytes [61]. Our data point to the novel finding of IRF5/TLR4 co-expression is induced by glucose fluctuations in THP-1 macrophages. These observations are congruent, at least in part, with increased expression of IRF5, TLR4, and critical endogenous TLR4 ligands found in murine model of systemic sclerosis, providing evidence that IRF5, after activation by TLR4, binds to the promoters of several key genes involved in the pathogenesis of systemic sclerosis [62]. Notably, activation of IRF-5 has been linked to all MyD88-restricted TLRs including the TLR4 [63]. Besides, hypoxic stress via induction of HIF-1α and inflammatory responses are also known to increase TLR4 expression in macrophages [64]. Nonetheless, the metabolic switching from oxidative to glycolytic metabolism during M2-to-M1 polarization may occur independently of HIF-1α [65].

Our data also show that exposure of THP-1 macrophages to RIH promoted the secretion of MMP-9 protein. MMP-9 is a pro-inflammatory protein belonging to a family of zinc-containing endoproteinases which are involved in cell migration, adhesion, apoptosis, and remodeling. In T2D patients, MMP-9 is involved in depletion of the components of extracellular matrix (ECM) and basement membranes, such as collagens, proteoglycans, elastin, laminin, fibronectin, and other glycoproteins [66]. MMP-9 is secreted by the activated polymorphonuclear leukocytes, and it leads to perpetuation of chronic inflammation and tissue destruction [67]. In line with our data showing hyperglycemia-induced MMP-9 secretion by macrophages, an association between blood glucose levels and MMP-9 was documented in patients with sepsis [68], acute ischemic stroke [69], acute coronary syndrome [70], and T2D [71]. MMP-9 is potentially involved in the pathogenesis and progression of obesity, atherosclerosis, cardiovascular disease, metabolic syndrome, and T2D, while the circulatory MMPs are potential biomarkers of cardiovascular disease in T2D patients [72]. Importantly, our data also show that IRF5 silencing in macrophages diminishes the RIH-induced MMP-9 production. It implies that IRF5 targeting may have significance for reducing metabolic inflammation and lowering risk for cardiovascular complications through the MMP-9-dependent mechanism. However, while commenting on the dynamic nature of macrophage polarity and their role in the biology of adipose tissue, Kraakman et al. argue that it is important to also consider a “non-glucose/insulin resistant-centric view” with regard to role of adipose tissue macrophages and that they could contribute significantly to the risk of other associated diseases such as cardiovascular disease and cancer [73].

The present data support for carrying out interventional studies with the aim to flatten the acute glucose fluctuations and to dampen the obesity/T2D-associated morbid changes. In addressing the potential existing inflammatory links between RIH and obesity/T2D, notably, only a few studies so far have explored effects of glycemic variability in truly normoglycemic individuals. Glucose fluctuations played a role in microvascular complications in T2D patients through stimulation of oxidative stress [74,75]. Consistent with these studies, glucose fluctuations were found to play a role in the development of microvascular disease, as suggested by data from human umbilical vein endothelial cells [76] and human retinal epithelial cells [77]. It was concluded that glucose fluctuations were more deleterious than consistently high glucose concentrations [77,78]. Several studies also point to an association between glycemic variability and cardiovascular disease [79,80]. High glucose variability was an independent predictor of poor vascular function and the data suggested that morbidly obese individuals with increased glucose fluctuations were at a greater risk of cardiovascular disease due to dysglycemia-induced oxidative stress and inflammation, even if they were normoglycemic by traditional criteria [81]. Similarly, daily glucose fluctuations or glucose swings, such as peaks and troughs, had a more potent triggering effect on oxidative stress than chronic sustained hyperglycemia [82]. Our data further corroborate that glycemic variability induces the M1 polarization and upregulation of inflammatory and cardiovascular disease markers in THP-1 macrophages.

However, our study involves a few shortcomings or caveats such as lack of additional inflammatory and cardiovascular markers data from primary human macrophages as well as the data depicting consequences of TLR4 silencing with or without IRF5 targeting. Unlike IRF5, activation of the IRF4 pathway induces an anti-inflammatory response as though both pathways share the MyD88 as common adapter protein required for TLR4-downstream signaling. Blocking the IRF5 pathway does not essentially promote the IRF4 pathway, suggesting that hypoglycemic conditions might trigger different pathways, which requires further investigations. It would also be interesting to see whether other patterns of glucose fluctuations could induce immunophenotypic and inflammatory changes in THP-1 monocytic cells and macrophages different than those herein presented. It also remains unclear whether hyperglycemia or glucose fluctuations promote the oxidative stress and reactive oxygen species (ROS) as an underlying mechanism of the TLR4-IRF5 upregulation in monocytes/macrophages. It would be of interest to see how the hypo- and hyperglycemic conditions affect the levels of oxidative stress and ROS in various cell types as well as how the IRF5 and IRF4 pathways crosstalk with other molecular components of respective signalosomes. All in all, further studies will be required to validate our preliminary findings and to address these concerns together with more research targeting the TLR4-IRF5 axis to evaluate its therapeutic significance in obesity/T2D.

In conclusion, we found that IRF5 upregulation in T2D patients’ monocytes was associated with indicators of hyperglycemia and inflammation. We further demonstrate that RIH or glucose fluctuations in vitro upregulate the expression of IRF5 together with markers of inflammation and the cardiovascular disease marker MMP-9. These data support the notion that controlling glucose fluctuations and/or IRF5 silencing could be beneficial to alleviate metabolic inflammation.

## Supplementary Materials

The following are available online at https://www.mdpi.com/2073-4409/9/8/1892/s1, Table S1: Demographic and study population characteristics, Table S2: Clinical characteristics of the study population.

## Abbreviations

ATCC	American type culture collection
BMI	Body mass index
CCR2	C-C motif chemokine receptor-2
DMEM	Dulbecco’s modified Eagle’s medium
FACS	Fluorescence-activated cell sorting
FBG	Fasting blood glucose
GAPDH	Glyceraldehyde 3-phosphate dehydrogenase
HbA1c	Glycated hemoglobin
HDL	High-density lipoprotein
HOMA-IR	Homeostatic model assessment of insulin resistance
ICAM-1	Intercellular adhesion molecule-1
IL-1β	Interleukin-1β
IRF5	Interferon regulatory factor-5
MCP-1	Monocyte chemoattractant protein-1
MMP-9	Matrix metalloproteinase-9
PMA	Phorbol 12-myristate 13-acetate
PRR	Pattern recognition receptors
RIH	Repetitive intermittent hyperglycemia
SARS-CoV-2	Severe acute respiratory syndrome coronavirus-2
siRNA	Small interfering RNA
T2D	Type-2 diabetes
TLR4	Toll-like receptor-4
TNF-α	Tumor necrosis factor-α
WMA	World medical association

## References

[1] Ceriello A., Ihnat M.A. (2010). ‘Glycaemic variability’: A new therapeutic challenge in diabetes and the critical care setting. Diabet. Med..

[2] Butler S.O., Btaiche I.F., Alaniz C. (2005). Relationship Between Hyperglycemia and Infection in Critically Ill Patients. Pharmacother. J. Hum. Pharmacol. Drug Ther..

[3] Ghazali N., O’brien J.M., Dungan K., Phillips G., Marsh C.B., Lemeshow S., Connors A.F., Preiser J.-C. (2008). Glucose variability and mortality in patients with sepsis*. Crit. Care Med..

[4] Huang J., Zhang X., Li J., Tang L., Jiao X., Lv X. (2014). Impact of glucose fluctuation on acute cerebral infarction in type 2 diabetes. Can. J. Neurol. Sci./J. Can. des Sci. Neurol..

[5] Yoo S., Lee H.-J., Lee H., Ryu H.G. (2017). Association Between Perioperative Hyperglycemia or Glucose Variability and Postoperative Acute Kidney Injury After Liver Transplantation. Anesthesia Analg..

[6] Kuroda M., Shinke T., Otake H., Sugiyama D., Takaya T., Takahashi H., Terashita D., Uzu K., Tahara N., Kashiwagi D. (2016). Effects of daily glucose fluctuations on the healing response to everolimus-eluting stent implantation as assessed using continuous glucose monitoring and optical coherence tomography. Cardiovasc. Diabetol..

[7] Torimoto K., Okada Y., Mori H., Tanaka Y. (2013). Relationship between fluctuations in glucose levels measured by continuous glucose monitoring and vascular endothelial dysfunction in type 2 diabetes mellitus. Cardiovasc. Diabetol..

[8] Hirakawa Y., Arima H., Zoungas S., Ninomiya T., Cooper M., Hamet P., Mancia G., Poulter N., Harrap S.B., Woodward M. (2014). Impact of Visit-to-Visit Glycemic Variability on the Risks of Macrovascular and Microvascular Events and All-Cause Mortality in Type 2 Diabetes: The ADVANCE Trial. Diabetes Care.

[9] Wu N., Shen H., Wang Y., He B., Zhang Y., Bai Y., Du R., Du Q., Han P. (2017). Role of the PKCβII/JNK signaling pathway in acute glucose fluctuation-induced apoptosis of rat vascular endothelial cells. Acta Diabetol..

[10] Azuma K., Kawamori R., Toyofuku Y., Kitahara Y., Sato F., Shimizu T., Miura K., Mine T., Tanaka Y., Mitsumata M. (2006). Repetitive Fluctuations in Blood Glucose Enhance Monocyte Adhesion to the Endothelium of Rat Thoracic Aorta. Arter. Thromb. Vasc. Biol..

[11] Barnes B., Lubyova B., Pitha P.M. (2002). Review: On the Role of IRF in Host Defense. J. Interf. Cytokine Res..

[12] Eguchi J., Yan Q.-W., Schones D.E., Kamal M., Hsu C.-H., Zhang M.Q., Crawford G.E., Rosen E.D. (2008). Interferon Regulatory Factors Are Transcriptional Regulators of Adipogenesis. Cell Metab..

[13] Sindhu S., Thomas R., Kochumon S., Wilson A., Abu-Farha M., Bennakhi A., Al-Mulla F., Ahmad R. (2019). Increased Adipose Tissue Expression of Interferon Regulatory Factor (IRF)-5 in Obesity: Association with Metabolic Inflammation. Cells.

[14] Sindhu S., Kochumon S., Thomas R., Bennakhi A., Al-Mulla F., Ahmad R. (2020). Enhanced Adipose Expression of Interferon Regulatory Factor (IRF)-5 Associates with the Signatures of Metabolic Inflammation in Diabetic Obese Patients. Cells.

[15] Kumari M., Wang X., Lantier L., Lyubetskaya A., Eguchi J., Kang S., Tenen D., Roh H.C., Kong X., Kazak L. (2016). IRF3 promotes adipose inflammation and insulin resistance and represses browning. J. Clin. Investig..

[16] Krausgruber T., Blazek K., Smallie T., Alzabin S., Lockstone H., Sahgal N., Hussell T., Feldmann M., Udalova I.A. (2011). IRF5 promotes inflammatory macrophage polarization and TH1-TH17 responses. Nat. Immunol..

[17] Wei J., Tang D., Lu C., Yang J., Lu Y., Wang Y., Jia L., Wang J., Ru W., Lu Y. (2019). Irf5 deficiency in myeloid cells prevents necrotizing enterocolitis by inhibiting M1 macrophage polarization. Mucosal Immunol..

[18] Castoldi A., De Souza C.N., Câmara N.O.S., Moraes-Vieira P.M.M. (2016). The Macrophage Switch in Obesity Development. Front. Immunol..

[19] Mori T., Anazawa Y., Iiizumi M., Fukuda S., Nakamura Y., Arakawa H. (2002). Identification of the interferon regulatory factor 5 gene (IRF-5) as a direct target for p53. Oncogene.

[20] Yanai H., Chen H.-M., Inuzuka T., Kondo S., Mak T.W., Takaoka A., Honda K., Taniguchi T. (2007). Role of IFN regulatory factor 5 transcription factor in antiviral immunity and tumor suppression. Proc. Natl. Acad. Sci. USA.

[21] Takaoka A., Yanai H., Kondo S., Duncan G., Negishi H., Mizutani T., Kano S.-I., Honda K., Ohba Y., Mak T.W. (2005). Integral role of IRF-5 in the gene induction programme activated by Toll-like receptors. Nature.

[22] Sindhu S., Kochumon S., Shenouda S., Wilson A., Al-Mulla F., Ahmad R. (2019). The Cooperative Induction of CCL4 in Human Monocytic Cells by TNF-α and Palmitate Requires MyD88 and Involves MAPK/NF-κB Signaling Pathways. Int. J. Mol. Sci..

[23] Ahmad R., Kochumon S., Chandy B., Shenouda S., Koshy M., Hasan A., Arefanian H., Al-Mulla F., Sindhu S. (2019). TNF-α Drives the CCL4 Expression in Human Monocytic Cells: Involvement of the SAPK/JNK and NF-κB Signaling Pathways. Cell. Physiol. Biochem..

[24] Kochumon S., Al-Roub A., Al-Ghanim M., Al-Mass A., Behbehani K., Ahmad R. (2015). TLR2 and AP-1/NF-kappaB are involved in the regulation of MMP-9 elicited by heat killed Listeria monocytogenes in human monocytic THP-1 cells. J. Inflamm..

[25] Ahmad R., Al-Roub A., Kochumon S., Akther N., Thomas R., Kumari M., Koshy M.S., Tiss A., Hannun Y.A., Tuomilehto J. (2018). The Synergy between Palmitate and TNF-α for CCL2 Production Is Dependent on the TRIF/IRF3 Pathway: Implications for Metabolic Inflammation. J. Immunol..

[26] Wray G.M., Foster S.J., Hinds C.J., Thiemermann C. (2001). A cell wall component from pathogenic and non-pathogenic Gram-positive bacteria (peptidoglycan) synergizes with endotoxin to cause the release of tumor necrosis factor-α, nitric oxide production, shock, and multiple organ injury/dysfunction in the rat. Shock.

[27] Al-Rashed F., Kochumon S., Usmani S., Sindhu S., Ahmad R. (2017). Pam3CSK4 Induces MMP-9 Expression in Human Monocytic THP-1 Cells. Cell. Physiol. Biochem..

[28] Ahmad R., El Bassam S., Cordeiro P., Menezes J. (2008). Requirement of TLR2-mediated signaling for the induction of IL-15 gene expression in human monocytic cells by HSV-1. Blood.

[29] Al-Rashed F., Alghaith A., Azim R., AlMekhled D., Thomas R., Sindhu S., Ahmad R. (2020). Increasing the Duration of Light Physical Activity Ameliorates Insulin Resistance Syndrome in Metabolically Healthy Obese Adults. Cells.

[30] Sindhu S., Al-Roub A., Koshy M., Thomas R., Ahmad R. (2016). Palmitate-Induced MMP-9 Expression in the Human Monocytic Cells is Mediated through the TLR4-MyD88 Dependent Mechanism. Cell. Physiol. Biochem..

[31] Thomas R., Al-Rashed F., Akhter N., Al-Mulla F., Ahmad R. (2019). ACSL1 Regulates TNFα-Induced GM-CSF Production by Breast Cancer MDA-MB-231 Cells. Biomolecules.

[32] Tirosh A., Shai I., Bitzur R., Kochba I., Tekes-Manova D., Israeli E., Shochat T., Rudich A. (2008). Changes in Triglyceride Levels Over Time and Risk of Type 2 Diabetes in Young Men. Diabetes Care.

[33] Stentz F.B., Kitabchi A.E. (2005). Hyperglycemia-induced activation of human T-lymphocytes with de novo emergence of insulin receptors and generation of reactive oxygen species. Biochem. Biophys. Res. Commun..

[34] Van Niekerk G., Davis T., Patterton H.-G., Engelbrecht A. (2019). How Does Inflammation-Induced Hyperglycemia Cause Mitochondrial Dysfunction in Immune Cells?. BioEssays.

[35] Joy N.G., Mikeladze M., Younk L.M., Tate D.B., Davis S.N. (2016). Effects of equivalent sympathetic activation during hypoglycemia on endothelial function and pro-atherothrombotic balance in healthy individuals and obese standard treated type 2 diabetes. Metabolism.

[36] Ratter J., Rooijackers H.M., Tack C.J., Hijmans A.G., Netea M.G., De Galan B.E., Stienstra R. (2017). Proinflammatory Effects of Hypoglycemia in Humans With or Without Diabetes. Diabetes.

[37] Dalmas E., Toubal A., Alzaid F., Blazek K., Eames H.L., Lebozec K., Pini M., Hainault I., Montastier E., Denis R. (2015). Irf5 deficiency in macrophages promotes beneficial adipose tissue expansion and insulin sensitivity during obesity. Nat. Med..

[38] Dalmas E., Venteclef N. (2015). Les macrophages-Nouveaux modulateurs de la répartition de la masse grasse au cours de l’obésité. Med. Sci. (Paris).

[39] Abu El-Asrar A.M. (2012). Role of Inflammation in the Pathogenesis of Diabetic Retinopathy. Middle East Afr. J. Ophthalmol..

[40] Reusch J.E., Wang C.C.L. (2011). Cardiovascular disease in diabetes: Where does glucose fit in?. J. Clin. Endocrinol. Metab..

[41] Drucker D.J., Sherman S.I., Gorelick F.S., Bergenstal R.M., Sherwin R.S., Buse J.B. (2010). Incretin-Based Therapies for the Treatment of Type 2 Diabetes: Evaluation of the Risks and Benefits. Diabetes Care.

[42] Kalra S., Mukherjee J.J., Ramachandran A., Saboo B., Shaikh S., Venkataraman S., Bantwal G., Das A.K. (2013). Hypoglycemia: The neglected complication. Indian J. Endocrinol. Metab..

[43] Grosick R., Alvarado-Vázquez P.A., Messersmith A.R., Romero-Sandoval E.A. (2018). High glucose induces a priming effect in macrophages and exacerbates the production of pro-inflammatory cytokines after a challenge. J. Pain Res..

[44] Pavlou S., Lindsay J., Ingram R.J., Xu H., Chen M. (2018). Sustained high glucose exposure sensitizes macrophage responses to cytokine stimuli but reduces their phagocytic activity. BMC Immunol..

[45] Nagareddy P.R., Kraakman M.J., Masters S.L., Stirzaker R.A., Gorman D.J., Grant R.W., Dragoljevic D., Hong E.S., Abdel-Latif A., Smyth S.S. (2014). Adipose Tissue Macrophages Promote Myelopoiesis and Monocytosis in Obesity. Cell Metab..

[46] Wentworth J.M., Naselli G., Brown W.A., Doyle L., Phipson B., Smyth G.K., Wabitsch M., O’Brien P.E., Harrison L.C. (2010). Pro-Inflammatory CD11c+CD206+ Adipose Tissue Macrophages Are Associated With Insulin Resistance in Human Obesity. Diabetes.

[47] Torres-Castro I., Arroyo-Camarena Ú.D., Martínez-Reyes C.P., Gómez-Arauz A.Y., Dueñas-Andrade Y., Hernández J., Béjar Y.L., Zaga-Clavellina V., Morales-Montor J., Terrazas L.I. (2016). Human monocytes and macrophages undergo M1-type inflammatory polarization in response to high levels of glucose. Immunol. Lett..

[48] Herder C., Müller-Scholze S., Rating P., Koenig W., Thorand B., Haastert B., Holle R., Illig T., Rathmann W., Seissler J. (2006). Systemic monocyte chemoattractant protein-1 concentrations are independent of type 2 diabetes or parameters of obesity: Results from the Cooperative Health Research in the Region of Augsburg Survey S4 (KORA S4). Eur. J. Endocrinol..

[49] Amano S.U., Cohen J.L., Vangala P., Tencerová M., Nicoloro S.M., Yawe J.C., Shen Y., Czech M.P., Aouadi M. (2013). Local proliferation of macrophages contributes to obesity-associated adipose tissue inflammation. Cell Metab..

[50] Weiss M., Blazek K., Byrne A.J., Perocheau D.P., Udalova I.A. (2013). IRF5 Is a Specific Marker of Inflammatory Macrophages In Vivo. Mediat. Inflamm..

[51] Dideberg V., Kristjansdottir G., Milani L., Libioulle C., Sigurdsson S., Louis E., Wiman A.-C., Vermeire S., Rutgeerts P., Belaiche J. (2007). An insertion deletion polymorphism in the Interferon Regulatory Factor 5 (IRF5) gene confers risk of inflammatory bowel diseases. Hum. Mol. Genet..

[52] Ciesla M., Kolarz B., Majdan M., Darmochwał-Kolarz D. (2019). IRF5 promoter methylation as a new potential marker of rheumatoid arthritis. Pol. Arch. Intern. Med..

[53] Lazzari E., Jefferies C.A. (2014). IRF5-mediated signaling and implications for SLE. Clin. Immunol..

[54] Barnes B.J., De S., Song S., Nelson V. (2018). IRF5 hyper-activation is a driver of systemic lupus erythematosus (SLE) onset and severity. J. Immunol..

[55] Corbin A.L., Gomez-Vazquez M., Berthold D.L., Attar M., Arnold I.C., Powrie F., Sansom S.N., Udalova I.A. (2020). IRF5 guides monocytes toward an inflammatory CD11c+ macrophage phenotype and promotes intestinal inflammation. Sci. Immunol..

[56] Krausgruber T., Saliba D., Ryzhakov G., Lanfrancotti A., Blazek K., Udalova I.A. (2010). IRF5 is required for late-phase TNF secretion by human dendritic cells. Blood.

[57] Laviada-Molina H.A., Leal-Berumen I., Rodriguez-Ayala E., Bastarrachea R.A. (2020). Working Hypothesis for Glucose Metabolism and SARS-CoV-2 Replication: Interplay Between the Hexosamine Pathway and Interferon RF5 Triggering Hyperinflammation. Role of BCG Vaccine?. Front. Endocrinol..

[58] Wang L., Wang J., Fang J., Zhou H., Liu X., Su S. (2015). High glucose induces and activates Toll-like receptor 4 in endothelial cells of diabetic retinopathy. Diabetol. Metab. Syndr..

[59] Shi H., Kokoeva M.V., Inouye K., Tzameli I., Yin H., Flier J.S. (2006). TLR4 links innate immunity and fatty acid–induced insulin resistance. J. Clin. Investig..

[60] Wang N., Liang H., Zen K. (2014). Molecular Mechanisms That Influence the Macrophage M1â€“M2 Polarization Balance. Front. Immunol..

[61] Dasu M.R., Devaraj S., Zhao L., Hwang D.H., Jialal I. (2008). High Glucose Induces Toll-Like Receptor Expression in Human Monocytes: Mechanism of Activation. Diabetes.

[62] Saigusa R., Asano Y., Taniguchi T., Yamashita T., Ichimura Y., Takahashi T., Toyama T., Yoshizaki A., Sugawara K., Tsuruta D. (2015). Multifaceted contribution of the TLR4-activated IRF5 transcription factor in systemic sclerosis. Proc. Natl. Acad. Sci. USA.

[63] Schoenemeyer A., Barnes B.J., Mancl M.E., Latz E., Goutagny N., Pitha P.M., Fitzgerald K.A., Golenbock D.T. (2005). The Interferon Regulatory Factor, IRF5, Is a Central Mediator of Toll-like Receptor 7 Signaling. J. Biol. Chem..

[64] Kim S.Y., Choi Y.J., Joung S.M., Lee B.H., Jung Y.-S., Lee J.Y. (2009). Hypoxic stress up-regulates the expression of Toll-like receptor 4 in macrophages via hypoxia-inducible factor. Immunology.

[65] Rodríguez-Prados J.-C., Traves P.G., Cuenca J., Rico D., Aragonés J., Martin-Sanz P., Cascante M., Boscá L. (2010). Substrate Fate in Activated Macrophages: A Comparison between Innate, Classic, and Alternative Activation. J. Immunol..

[66] Mun-Bryce S., Rosenberg G.A. (1998). Matrix Metalloproteinases in Cerebrovascular Disease. Br. J. Pharmacol..

[67] Khokha R., Murthy A., Weiss A. (2013). Metalloproteinases and their natural inhibitors in inflammation and immunity. Nat. Rev. Immunol..

[68] Sachwani G.R., Jaehne A.K., Jayaprakash N., Kuzich M., Onkoba V., Blyden D., Rivers E.P. (2016). The association between blood glucose levels and matrix-metalloproteinase-9 in early severe sepsis and septic shock. J. Inflamm..

[69] Setyopranoto I., Malueka R.G., Panggabean A.S., Widyadharma I.P.E., Sadewa A.H., Lamsudin R., Wibowo S. (2018). Association between Increased Matrix Metalloproteinase-9 (MMP-9) Levels with Hyperglycaemia Incidence in Acute Ischemic Stroke Patients. Open Access Maced. J. Med. Sci..

[70] Setianto B.Y., Achmad A.F., Purnomo L.B. (2014). Serum matrix metalloproteinase-9 levels in acute coronary syndrome patients with and without hyperglycemia. Acta Med. Indones..

[71] Uemura S., Matsushita H., Li W., Glassford A.J., Asagami T., Lee K.H., Harrison D.G., Tsao P.S. (2001). Diabetes mellitus enhances vascular matrix metalloproteinase activity: Role of oxidative stress. Circ. Res..

[72] Ruiz-Ojeda F.J., Méndez-Gutiérrez A., Aguilera C.M., Plaza-Diaz J. (2019). Extracellular Matrix Remodeling of Adipose Tissue in Obesity and Metabolic Diseases. Int. J. Mol. Sci..

[73] Kraakman M.J., Murphy A.J., Jandeleit-Dahm K., Kammoun H.L. (2014). Macrophage Polarization in Obesity and Type 2 Diabetes: Weighing Down Our Understanding of Macrophage Function?. Front. Immunol..

[74] Brownlee M. (2005). The Pathobiology of Diabetic Complications: A Unifying Mechanism. Diabetes.

[75] Brownlee M., Hirsch I.B. (2006). Glycemic Variability: A Hemoglobin A1c–Independent Risk Factor for Diabetic Complications. JAMA.

[76] Quagliaro L., Piconi L., Assaloni R., Martinelli L., Motz E., Ceriello A. (2003). Intermittent high glucose enhances apoptosis related to oxidative stress in human umbilical vein endothelial cells: The role of protein kinase C and NAD(P)H-oxidase activation. Diabetes.

[77] Sun J., Xu Y., Sun S., Sun Y., Wang X. (2010). Intermittent high glucose enhances cell proliferation and VEGF expression in retinal endothelial cells: The role of mitochondrial reactive oxygen species. Mol. Cell. Biochem..

[78] Jones S.C., Saunders H.J., Qi W., Pollock C.A. (1999). Intermittent high glucose enhances cell growth and collagen synthesis in cultured human tubulointerstitial cells. Diabetologia.

[79] Hermanides J., Vriesendorp T.M., Bosman R.J., Zandstra D.F., Hoekstra J.B., Devries J.H. (2010). Glucose variability is associated with intensive care unit mortality*. Crit. Care Med..

[80] Hu Y., Liu W., Huang R., Zhang X. (2010). Postchallenge plasma glucose excursions, carotid intima-media thickness, and risk factors for atherosclerosis in Chinese population with type 2 diabetes. Atherosclerosis.

[81] Buscemi S., Re A., Batsis J.A., Arnone M., Mattina A., Cerasola G., Verga S. (2010). Glycaemic variability using continuous glucose monitoring and endothelial function in the metabolic syndrome and in Type 2 diabetes. Diabet. Med..

[82] Monnier L., Mas E., Ginet C., Michel F., Villon L., Cristol J.-P., Colette C. (2006). Activation of Oxidative Stress by Acute Glucose Fluctuations Compared With Sustained Chronic Hyperglycemia in Patients With Type 2 Diabetes. JAMA.

