# Supplementary material for: Repetitive Intermittent Hyperglycemia Drives the M1 Polarization and Inflammatory Responses in THP-1 Macrophages Through the Mechanism Involving the TLR4-IRF5 Pathway
Source: Cells. 2020 Aug 12;9(8):1892. doi: 10.3390/cells9081892 (PMC7463685; doi:10.3390/cells9081892)
Supplement: Supplementary file 1 [file cells-09-01892-s001.pdf]

Table 1. Demographic and study population characteristics.

Physical characteristics of subjects	Nondiabetic group (N = 48)	Diabetic group (N = 39)	*p-Value*
Age (years)	44.8 ± 12.1	49.5 ± 9.6	0.380
Weight (KG)	80.1 ± 11.1	83.4 ± 11.6	0.063
Height (CM)	1.64 ± 0.1	1.65 ± 0.08	0.539
BMI (KG/M2)	28.0 ± 6.1	30.2 ± 4.3	0.065
Waist to Hip circumference	0.89 ± 15.5	0.91 ± 8.2	0.323
Body fat percentage (%)	32.2 ± 8.5	35,2 ± 6.6	0.133

All values are means ± standard deviations unless labeled otherwise.

Table 2. Clinical characteristics of the study population.

Fasting concentration of serum sample	Nondiabetic group (N = 48)	Diabetic group (N = 39)	*p-Value*
Fasting glucose (mmol/l)	4.7 ± 0.3	6.6 ± 1.3	**<0.0001**
Triglycerides (mmol/l)	0.91 ± 0.7	1.6 ± 1.0	**<0.0001**
Total cholesterol (mmol/l)	4.7 ± 0.8	5.11 ± 1.5	0.124
HDL cholesterol (mmol/l)	1.33 ± 0.3	1.25 ± 0.6	0.489
Insulin Con. (mu/l)	9.8 ± 8.3	25.3 ± 10.8	**0.0006**
HOMA-IR	2.8 ± 1.9	6.7 ± 4.3	**0.042**

All values are means ± standard deviations unless labeled otherwise.
